# A Comparative Study of the Antioxidant Status and Biotechnological Potential of *Bracteacoccus minor* (Chlorophyceae) Strains

**DOI:** 10.3390/ijms262110740

**Published:** 2025-11-05

**Authors:** Irina Maltseva, Angelica Kochubey, Aleksandr Yakoviichuk, Svetlana Maltseva, Maxim Kulikovskiy, Yevhen Maltsev

**Affiliations:** 1 Faculty of Natural Sciences, Melitopol State University, 72312 Melitopol; maltseva-irina22@yandex.ru (I.M.); kochubeya92@yandex.ru (A.K.); alex.yakov1991@gmail.com (A.Y.); 2 K.A. Timiryazev Institute of Plant Physiology RAS, IPP RAS, 127276 Moscow, Russia; svetadm32@gmail.com (S.M.); max-kulikovsky@yandex.ru (M.K.)

**Keywords:** antioxidant enzymes, α-tocopherol, carotenoids, chlorophyll, fatty acid, lipid peroxidation

## Abstract

The accumulation of metabolites and the antioxidant response in new strains of *Bracteacoccus minor* MZ–Ch31 and MZ–Ch39 isolated from various biotopes were studied. It was found that the antioxidant response of *B*. *minor* MZ–Ch31 and MZ–Ch39 is different. This may be due to the acclimatization of these strains to specific environmental factors of the natural biotope. Strain *B*. *minor* MZ–Ch39 had a higher antioxidant activity compared to *B*. *minor* MZ–Ch31. The TBA-reactive substance content in *B*. *minor* MZ–Ch39 cells was lower than that of MZ–Ch31. The antioxidant response in *B*. *minor* MZ–Ch39 was realized by high catalase and glutathione peroxidase activity and accumulation of retinol. In *B*. *minor* MZ–Ch31, the antioxidant response was associated with the accumulation of α-tocopherol and carotenoids. The strains did not differ in terms of superoxide dismutase activity. From a biotechnological point of view, *B. minor* MZ–Ch31 biomass is a valuable resource of lipids rich in omega-3 fatty acids, α-tocopherol, and carotenoids. The *B. minor* MZ–Ch39 has the potential to generate lipids enriched with essential omega-6 fatty acids.

## 1. Introduction

Numerous studies have demonstrated that microalgae biomass is rich in a variety of biologically active compounds. These compounds have the potential to improve the quality of various products, including food, feed, cosmetics, and medical products [1,2,3].

Microalgae can grow in bioreactors, which can be located in desert areas where conventional crops do not grow. This allows us to obtain additional products and overcome limitations on arable land. The wide variety of microalgae species, combined with their distinctive biochemical properties, represents a valuable natural resource that is currently undergoing active research. To improve the profitability of microalgae-based products, scientists are searching for new, highly productive strains of algae in their natural habitats [4,5]. The effectiveness of using molecular and metabolic engineering techniques and gene editing to induce the production of biologically active compounds is currently being investigated [6,7], Additionally, technologies for algae cultivation under abiotic stress conditions are being developed [8,9].

It is known that metabolic changes in cells under stress initiate an increase in the production of reactive oxygen species (ROS). These ROS initiate the lipid peroxidation process (LPO) [10,11,12,13,14], or antioxidant response formation. The antioxidant reaction triggers a variety of changes in cellular systems, including those related to antioxidants, energy, photosynthesis, proteins, lipids, fatty acids (FA), and the production of secondary metabolites [15,16,17,18,19,20].

Many secondary metabolites that are useful in biotechnology have antioxidant properties and are part of the antioxidant defense system (AOS). They are one of the substances that neutralize ROS, which consequently leads to their depletion during the production of large amounts of ROS. In addition, alternative mechanisms for increasing antioxidant resistance in cells include regulating the activity of enzymes involved in energy metabolism, which generate ROS. This also involves reducing the content of unsaturated fatty acids, which are the primary substrate for LPO.

Stress management may become a promising technology for achieving the best microalgae productivity in biotechnological industries. However, modern research suggests that the mechanisms of cellular regulation and stress adaptation in microalgae are complex, and scientists have yet to investigate them thoroughly [21,22]. Most research focuses on the study of individual antioxidants, primarily enzymes, under the influence of various stressors [23,24,25]. The revealed patterns of the antioxidant reaction of microalgae are still ambiguous. The antioxidant response in different species and even strains of the same species to the action of an identical stress factor may not coincide, and the cell can achieve redox balance by regulating the content of various antioxidants and activating various enzymes [22,26,27].

A comprehensive study of low-molecular antioxidants, the fatty acid composition, energy system state, and the activity of antioxidant enzymes can provide valuable insights into the direction of metabolic restructuring, as well as the contribution of each component to the antioxidant response. A comparative analysis of these indicators will provide new information on the functioning of antioxidant protection in microalgae and identify stress-resistant strains of microalgae for further biotechnological applications. In addition, based not only on the quantitative content of target products but also on the nature of their contribution to the formation of an antioxidant response, we can determine which products will accumulate under the influence of a particular stressor. This allows us to assess the strain’s resistance to the intensity and duration of stressor effects. In the future, this could allow us to switch to techniques for controlling stress and interrupting it at the peak concentration of target metabolites.

Green microalgae from the Sphaeropleales are known for their high potential in biotechnology. Many scientists have reported that high amounts of lipids and carotenoids accumulate in the various representatives of the Sphaeropleales: *Coelastrella* Chodat [28,29], *Scenedesmus* Meyen, *Dictyochloris* Vischer [30,31,32], *Monoraphidium* Komarkova-Legnerova, *Pseudomuriella* N. Hanagata [33,34], *Nephrochlamys* Korshikov [35]. Recently, there has been a lot of attention paid to the study of the biotechnological potential of the *Bracteacoccus* Tereg [5,36]. There is evidence that *Bracteacoccus* produces large amounts of carotenoids, lipids, and valuable fatty acids [5,37,38,39,40,41]. *Bracteacoccus* species live worldwide in a variety of habitats, including forests and desert soils, caves, mountains, snow-covered areas, and aquatic environments [5,36,39,41,42,43]. Ecological plasticity indicates the presence of a broad range of metabolic abilities in *Bracteacoccus*, which increases interest in finding and studying new high-performing and biotechnologically valuable species or strains from various natural habitats. When studying the species diversity of algae in forest soil, two strains were isolated: MZ–Ch31 and MZ–Ch39. Their morphological features correspond to *B. minor* (Schmidle ex Chodat) Petrová. Molecular studies of the variable region V4 of the 18S rRNA gene and the chloroplast *rbc*L gene (not published) have confirmed this. The strain *B. minor* MZ–Ch31 was present in the soil of *Robinia pseudoacacia* L. planting in the Zaporozhye region, and *B. minor* MZ–Ch31 was also in the soils of *Pinus sylvestris* L. forest in the Voronezh region.

At the moment, there is very little information available about the biotechnology aspects of soil *B. minor*. Chubchikova et al. [44] and Minyuk et al. [38] reported that the strain *B. minor* ACKU 506-06 ( = SAG 221-1) could accumulate lipids and carotenoids. Despite the limited number of studies on the biochemical parameters of *B. minor*, the literature has described the ability of this genus to accumulate high levels of lipids, carotenoids, and essential fatty acids in response to various stress types [5,36,37,38,39,40,41,43,45,46,47]. This suggests the potential for further research into various *Bracteacoccus* species and the improvement of their production characteristics through the use of stress. At the moment, there is not enough information in the literature to establish patterns of metabolite accumulation and antioxidant response formation in *Bracteacoccus* species. The study by Santhakumaran et al. [48] mentions the antioxidant resistance of *B*. *minor*. However, a comprehensive study of the current state of AOS and the content of various antioxidants is a priority in the strategy for selecting strains for further biotechnological use. This approach has been applied by several scientists [49,50,51].

In this study, we employed an integrated approach and studied the molecular-phylogenetic, biochemical features and specificity of the antioxidant response, and accumulation of metabolites in new *Bracteacoccus* strains isolated from various biotopes, for assessing their biotechnological potential.

## 2. Results

### 2.1. Strains Description

**Figure 1:**
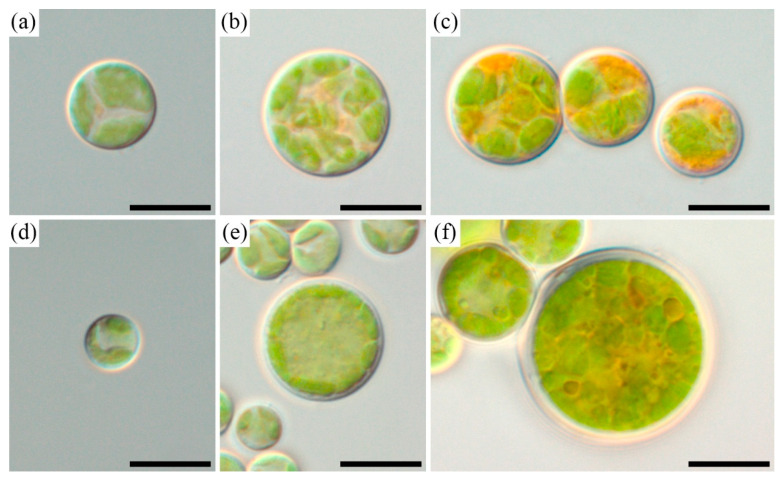
Light micrograph gallery of representatives of *Bracteacoccus minor* cells: young vegetative cell, 2 weeks of age, MZ–Ch31 (**a**), mature vegetative cell, 4 weeks of age, MZ–Ch31 (**b**), mature vegetative cell with orange oil droplets, 8 weeks of age, MZ–Ch31 (**c**), young vegetative cell, 1 week of age, MZ–Ch39 (**d**), mature vegetative cell, 4 weeks of age, MZ–Ch39 (**e**), mature vegetative cell with yellow-orange oil droplets, 8 weeks of age, MZ–Ch39 (**f**). Scale bar 10 μm.

*Bracteacoccus minor* MZ–Ch31 (Chlorophyceae, Sphaeropleales) (Figure 1a–c).

MORPHOLOGICAL DESCRIPTION: Vegetative cells spherical, 8.5–18 μm in diameter. Cell wall thin and smooth. Chloroplast single in young cells, bilobed, parietal. Chloroplasts numerous in mature cells, lamellar, spherical, and sometimes irregular; pyrenoid-free. Older cells accumulate orange oil droplets. Asexual reproduction by aplanospore formation. Aplanospore 4–5 μm in diameter. Sexual reproduction not known.

HABITAT: The strain was isolated from a sample of the upper 5 cm layer of soil, taken in the *Robinia pseudoacacia* plantation with sparse grass coverage and an underdeveloped forest litter at M. Gorky Melitopol Central Park of Culture and Recreation (N 46°837631, E 35°362555), Melitopol, Zaporizhzhia region, 10 November 2012. The soil is kastanozem; the pH (water) of the soil is 5.84; the humus content is 5.9%; and the ash content is 88.92%.

SEQUENCE DATA: GenBank accession PX426839 for the 18S rRNA gene partial sequence.

*Bracteacoccus minor* MZ–Ch39 (Chlorophyceae, Sphaeropleales) (Figure 1d–f).

MORPHOLOGICAL DESCRIPTION: Vegetative cells spherical, 10–26 μm in diameter. Cell wall thin, but may reach 1.5 μm in thickness after 6 months of growth. Chloroplast single in young cells, bilobed, parietal. Chloroplasts numerous in mature cells, spherical or plate-like, and sometimes irregular; pyrenoid-free. Older cells accumulate yellow-orange oil droplets. Asexual reproduction by aplanospore formation. Aplanospore 4.5–5.5 μm in diameter. Sexual reproduction not known.

HABITAT: The strain was isolated from a sample of the upper 5 cm layer of soil, taken in *Pinus sylvestris* forest with well-developed undergrowth and a developed forest litter (N 51°899596, E 39°431821), Ramon, Voronezh region, 9 October 2015. The soil is gleysols; the pH (water) of the soil is 5.2; the humus content is 15.7%; and the ash content is 68.5%.

SEQUENCE DATA: GenBank accession PX426840 for the 18S rRNA gene partial sequence.

**Figure 2:**
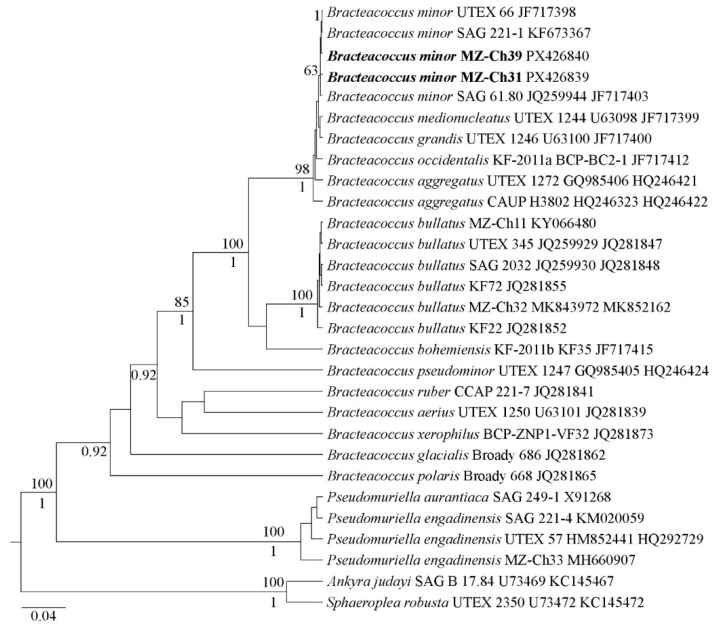
Phylogenetic position of new *Bracteacoccus* species (indicated in bold) based on Bayesian inference from an alignment of 29 sequences and 1,103 characters (18S rRNA gene and ITS1–5.8S rDNA–ITS2 region). Values above the horizontal lines are bootstrap support from ML analyses (values below 50 are not shown). Values under the horizontal lines (or to the right of the slash) are Bayesian posterior probabilities (values below 0.9 are not shown). Strain numbers (if available) and NCBI database accession numbers are indicated for all sequences.

MOLECULAR ANALYSIS: Phylogenetic analysis with maximum likelihood (ML) and Bayesian inference (BI) methods shows that MZ–Ch31 and MZ–Ch39 with *B*. *aggregatus* Tereg, *B*. *bullatus* Fučíková, Flechtner et Lewis, *B*. *bohemiensis* Fučíková, Flechtner et Lewis, *B. minor* (=*B*. *grandis* Bischoff et Bold, =*B*. *medionucleatus* Bischoff et Bold) and *B*. *occidentalis* Fučíková, Flechtner et Lewis strains formed a unified clade with high statistical support (likelihood bootstrap 98 and posterior probability 1.0) within Sphaeropleales. The tree topology is consistent with previous studies [36,39,52]. Figure 2 shows that strains MZ–Ch31 and MZ–Ch39 are close relatives of *B*. *minor*, including the type strain of *B*. *minor*, UTEX 66.

According to the results of morphological and phylogenetic analyses, the studied strains MZ–Ch31 and MZ–Ch39 are *B. minor*.

### 2.2. Biomass Dry Weight Content

**Figure 3:**
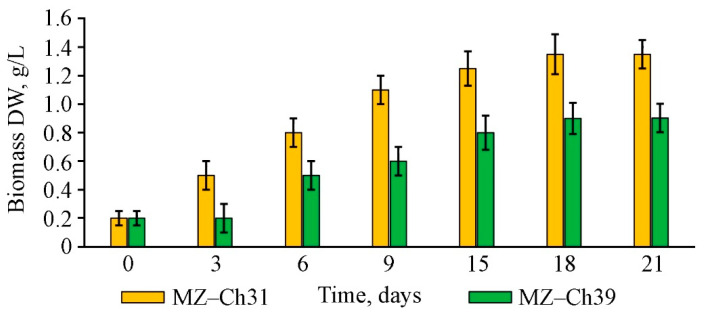
Dry biomass content in cell culture of *B. minor* MZ–Ch31, *B. minor* MZ–Ch39 (M ± SD, n = 3).

The dry biomass content in the culture of *B. minor* MZ–Ch31 on the 21st day of cultivation was 1.35 ± 0.10 g/L, 49.5% higher than *B. minor* MZ–Ch39 (Figure 3). The lag phase in *B. minor* MZ–Ch39 relative to *B. minor* MZ–Ch31 was more prolonged and amounted to 3 days. Three days later, *B. minor* MZ–Ch31 showed a 2.5-fold dry biomass increase. The log-growth phase for both strains lasted 18 days, after which the biomass content did not increase significantly.

### 2.3. Chlorophyll Content

**Figure 4:**
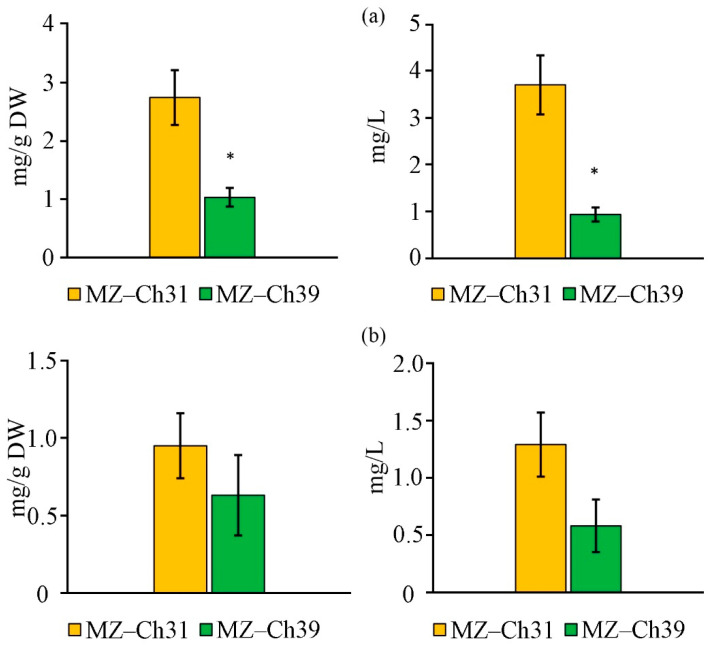
Chlorophyll content on the dry biomass and cell culture of *B. minor* MZ–Ch31, *B. minor* MZ–Ch39: chlorophyll *a* (**a**), chlorophyll *b* (**b**). Note. Here and further on in Figure 5, Figure 6, Figure 7, Figure 8 and Figure 9: medium BBM; culture volume 150 mL; cultivation time 21 days; M ± SD, n = 3; * the differences are significant relative to *B. minor* MZ–Ch31 (*p* ≤ 0.05).

The screening of isolates for chlorophyll content revealed 2.7-fold and 1.5-fold higher levels of Chl *a* and Chl *b*, respectively, in *B. minor* MZ–Ch31 compared to the strain *B. minor* MZ–Ch39 of dry biomass (DW) (Figure 4). The volume concentrations of Chl *a* and Chl *b* in the culture of *B. minor* MZ–Ch31 were 3.70 ± 0.63 and 1.29 ± 0.28 mg/L, respectively, while the concentrations of these pigments were 0.93 ± 0.15 and 0.58 ± 0.23 mg/L in *B. minor* MZ–Ch39.

### 2.4. Secondary Metabolites Content

**Figure 5:**
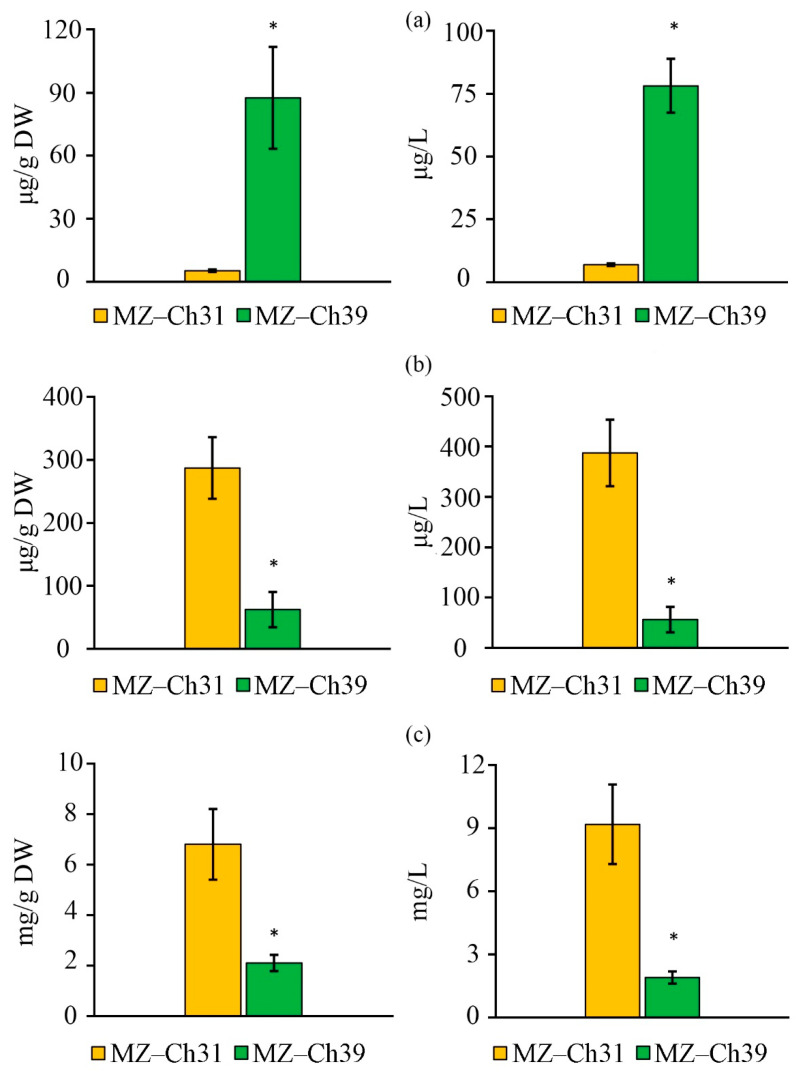
Fatty-soluble vitamins and carotenoids content on the dry biomass and cell culture of *B. minor* MZ–Ch31, *B. minor* MZ–Ch39: retinol (**a**), α-tocopherol (**b**), carotenoids (**c**). An asterisk indicates a significant difference compared with the control (*p* < 0.05, paired two-sample *t*-test).

The dry biomass of *B. minor* MZ–Ch31 has 16.9 times less retinol content than *B. minor* MZ–Ch39, and volume concentration is 11.2 times lower (Figure 5a).

The α-tocopherol content in *B. minor* MZ–Ch31 was 287.70 ± 10.49 μg/g DW and 387.50 ± 16.20 μg/L, which is 4.6 and 6.9 times greater than the content found in *B. minor* MZ–Ch39, respectively (Figure 5b).

The carotenoid content in the dry biomass of *B. minor* MZ–Ch31 was 3.2 times higher than that in *B. minor* MZ–Ch39 (Figure 5c). The volume concentration of carotenoids in *B. minor* MZ–Ch31 is 4.9 times higher than in *B. minor* MZ–Ch39.

### 2.5. Lipid Content

**Figure 6:**
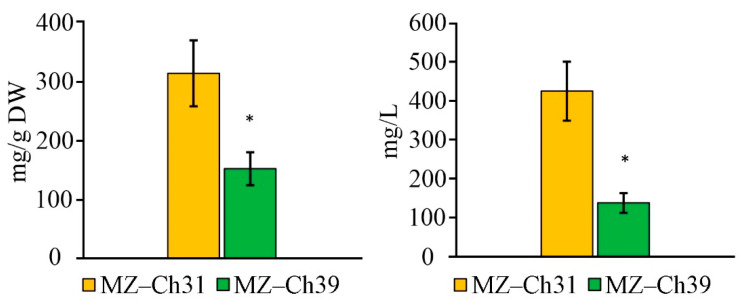
Lipid content in the dry biomass and cell culture of *B. minor* MZ–Ch31, *B. minor* MZ–Ch39. An asterisk indicates a significant difference compared with the control (*p* < 0.05, paired two-sample *t*-test).

The lipid content of the strain *B. minor* MZ–Ch31 was 314.0 ± 11.56 mg/g DW, which is 2.1 times higher than the total lipid content for the *B. minor* MZ–Ch39 biomass (Figure 6). In volume terms, the lipid content in the biomass of *B. minor* MZ–Ch31 was 3.1 times higher than that of *B. minor* MZ–Ch39.

### 2.6. Lipid Peroxidation Substances

**Figure 7:**
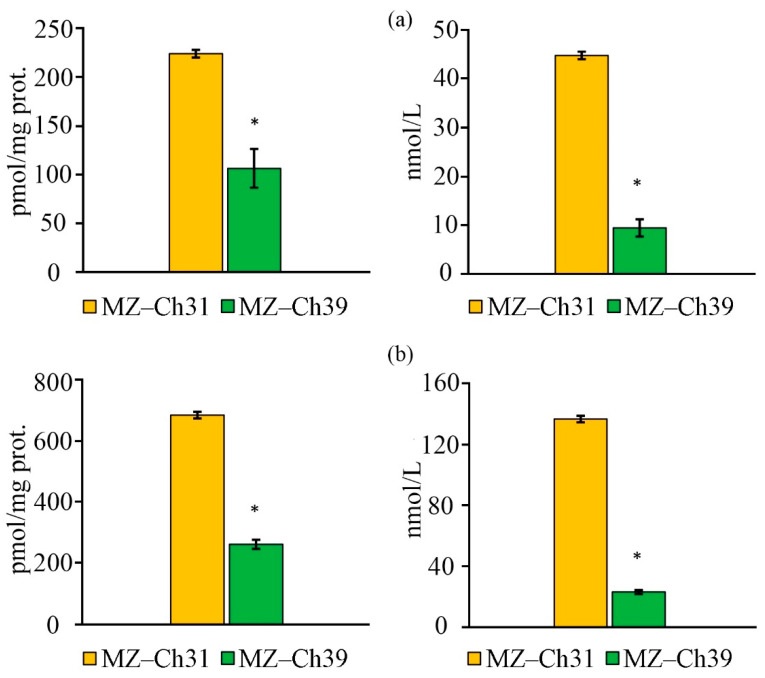
TBA-reactive substances content on the protein and cell culture of *B. minor* MZ–Ch31, *B. minor* MZ–Ch39: TBARS (**a**), TBARS_in_ (**b**). An asterisk indicates a significant difference compared with the control (*p* < 0.05, paired two-sample *t*-test).

The TBA-reactive substances (TBARS) concentration of *B. minor* MZ–Ch31 is 2.1 times higher than that of *B. minor* MZ–Ch39 (Figure 7a).

After the initiation of LPO by Fe^2+^ ions, the TBARS_in_ content in *B. minor* MZ–Ch31 is 2.6 times higher compared to *B. minor* MZ–Ch39 (Figure 7b). The volume content of TBARS and TBARS_in_ in the strain *B. minor* MZ–Ch39 is 78.9% and 83.0% lower than that of the strain *B. minor* MZ–Ch31.

### 2.7. Antioxidant Enzyme Activity

**Figure 8:**
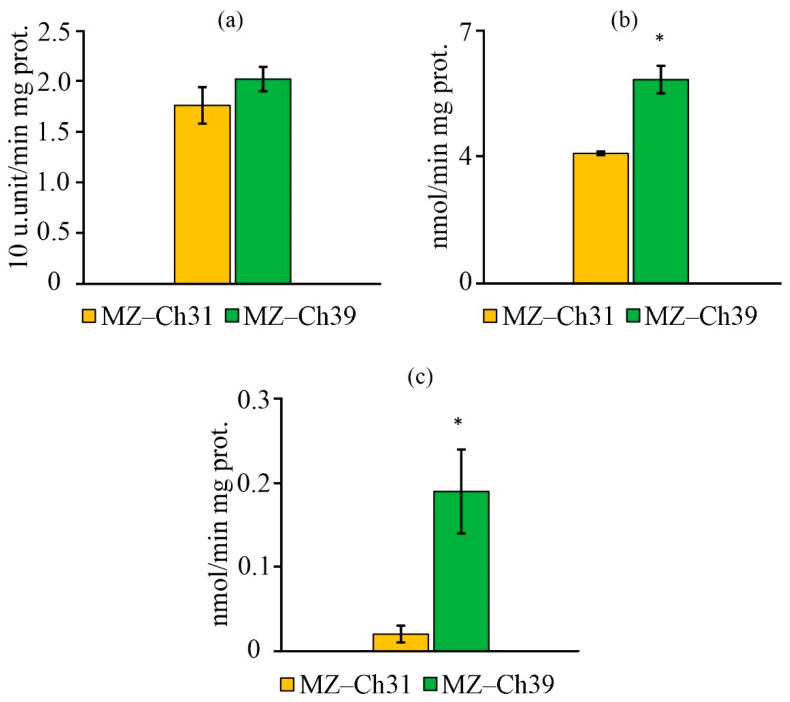
Antioxidant enzymes activity on the protein and cell culture of *B. minor* MZ–Ch31, *B. minor* MZ–Ch39: SOD-activity (**a**), CAT-activity (**b**), GPx-activity (**c**). An asterisk indicates a significant difference compared with the control (*p* < 0.05, paired two-sample *t*-test).

For the biomass of *B. minor* MZ–Ch31, the superoxide dismutase (SOD) activity did not significantly differ between the studied strains (Figure 8a). The specific catalase (CAT) activity in the biomass of *B. minor* MZ–Ch31 is 1.6 times lower, and the glutathione peroxidase (GPx) activity is 9.5 times higher compared to the strain *B. minor* MZ–Ch39 (Figure 8b,c).

### 2.8. Fatty Acids Profile, Unsaturation Index and Antioxidant Activity Coefficient

The lipid FA composition of *B. minor* MZ–Ch31 and *B. minor* MZ–Ch39 differed significantly in qualitative and quantitative terms (Table 1).

The strain *B. minor* MZ–Ch31 has 11.65% more saturated fatty acids (SFA) and 14.33% omega-3 fatty acids compared to the strain *B. minor* MZ–Ch39. The mass fraction of monounsaturated (MUFA) and omega-6 fatty acids in *B. minor* MZ–Ch39 is 2.5 and 1.9 times higher, respectively, compared to the strain *B. minor* MZ–Ch31. The total content of polyunsaturated fatty acids (PUFA) was not significantly different between the strains.

**Figure 9:**
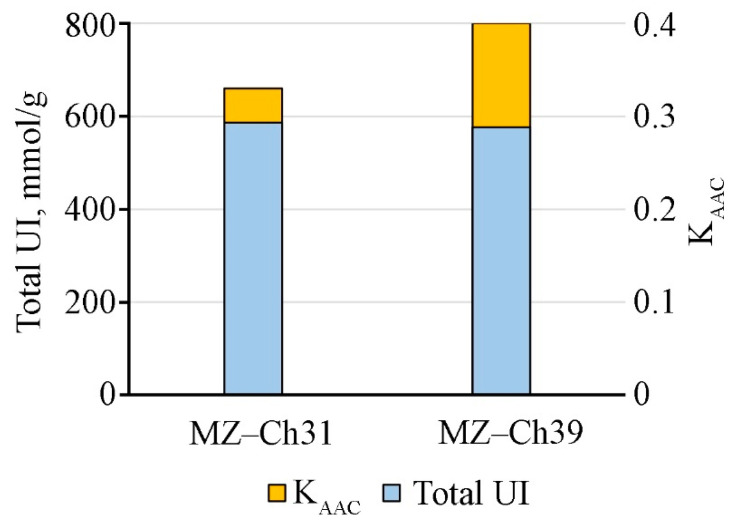
Antioxidant activity coefficient and total fatty acids unsaturation index of strains *B. minor* MZ–Ch31, *B. minor* MZ–Ch39.

These changes are due to differences in the composition of individual fatty acids. In particular, there was a 71.2% and 91.6% increase in the concentration of stearic 18:0 and α-linolenic 18:3n-3 acids in *B. minor* MZ–Ch31 compared to *B. minor* MZ–Ch39. *B. minor* MZ–Ch39 has concentrations of oleic 18:1n-9, hexadecadienoic 16:2n-6, and linoleic 18:2n-6 acids 3.1, 2.4, and 1.7 times higher than *B. minor* MZ–Ch39, respectively. There are no long-chain saturated lignoceric 24:0 and serosinic 26:0 acids in the biomass of *B. minor* MZ–Ch39. The unsaturation index of fatty acids was not different between the studied strains (Figure 9).

The antioxidant activity coefficient of the strain *B. minor* MZ–Ch31 is 17.5% lower than that of the strain *B. minor* MZ–Ch39 (Figure 9).

## 3. Discussion

### 3.1. Biomass Dry Weight Content

The strains *B*. *minor* MZ–Ch31 and *B*. *minor* MZ–Ch39 showed comparable or increased biomass density among representatives of *Bracteacoccus* [44,45]. The exceptions are *B. minor*, which grew in an environment with a high nitrogen content [38], and *B. bullatus*, which grew at high temperature and light intensity [5]. In this context, the strain *B. minor* MZ–Ch31 with a biomass density of 1.35 ± 0.10 g/L looks promising from the point of view of biotechnology.

*B. minor* MZ–Ch31 and *B. minor* MZ–Ch39, being representatives of the same species, showed almost a 50% difference in biomass concentration on 21 days of cultivation. This may be due to environmental factors at the site of strain isolation. This fact becomes obvious when comparing the results with the literature data. However, most of the literature data presented are for microalgae crops grown under conditions different from those used by us. In general, the biomass density for representatives of *Bracteacoccus* varied in the range of 0.30–2.88 g/L. The strain *B. minor* SAG 221-1 (=CCAP 221/1, =UTEX 66, =ACKU 506-06, =IBSS-88) isolated from the soil of a spruce forest (Belgium, Haute Ardenne) on the 16th day of cultivation reached a biomass density of 2.50 g/L, which exceeds similar indicators of the studied strains. But the strain was grown on 3N BBM medium enriched with nitrogen. When diluting the culture, the biomass concentration after 12 days of cultivation was only 1.10 g/L [38], which is lower than the characteristics of the strain *B. minor* MZ–Ch31. When the cultivation conditions change, the same *B*. *minor* SAG 221-1 (=CCAP 221/1, =UTEX 66, =ACKU 506-06, =IBSS-88) and the soil strain *B*. *giganteus* H.W.Bischoff et H.C.Bold ACKU 461-06 (=IBSS-87), grown on 3N BBM with a further 10-fold dilution (medium containing acetate (0.05 M) and sodium chloride (0.2 M)), after 14 days of cultivation, the biomass density was 0.46–0.50 g/L, respectively [44]. The soil strain *Bracteacoccus* sp. MIC-G16 (India, Rohtang Pass, mountains) reached a density of 0.58 g/L after 18 days when cultivated on BBM medium, and densities of 0.90 and 0.30 g/L when cultivated under mixotrophic and heterotrophic conditions, respectively [45]. The freshwater strain *B. pseudominor* BERC09 (municipal wastewater, Faisalabad, Punjab, Pakistan) reached a biomass density of 0.77–0.81 g/L under optimal lighting conditions at BBM [41], and the strain *B. aggregatus* BM5/15 (= IPPAS C-2045) isolated from the White Sea after 14 days of cultivation on a standard medium reached a concentration of 1.10 g/L [40]. The epiphytic strain *B. bullatus* CCALA 1120 isolated from the snow surface at optimally selected temperature and lighting reached a biomass density of 2.88 g/L [5].

The wide range of variation in biomass density among strains within the species suggests a significant influence of environmental factors on biomass density and the rate of its accumulation.

### 3.2. Chlorophyll Content

Photosynthesis is a crucial process in microalgal cells and it is sensitive to various stressors [53].

Changes in the photosynthetic apparatus occur relatively quickly under negative influence and are one of the early stages in the development of the general adaptation syndrome. Many authors have shown that abiotic stressors cause changes in the concentration and ratio of photosynthetic pigments [54,55,56]. This ratio, between different pigments, is considered to be a biological marker for the physiological state of plants. Photosynthesis proceeds with the absorption of carbon dioxide and the biosynthesis of primary and secondary metabolites [57]. Most of these metabolites have a significant effect on the regulation of photosynthesis in microalgal cells [53], and they provide antioxidant protection by neutralizing ROS [19]. In this case, the higher chlorophyll *a* content of *B. minor* MZ–Ch31 compared to *B. minor* MZ–Ch39 indicates increased metabolic and photosynthetic activity in the former. This is because chlorophyll *a* is the main component of light-harvesting complexes in chloroplasts. It is responsible for capturing light energy and converting it into chemical energy during photosynthesis. Active photosynthesis is associated with the generation of significant amounts of free radicals [58,59,60] and an increase in LPO, which confirms the accumulation of TBARS and TBARS_in_. As a result, the *B. minor* MZ–Ch31 variety contained higher concentrations of carotenoids and α-tocopherol, which act as antioxidants and protect cells from oxidative damage.

The example of *Chlorella* spp. showed that the ratio of chlorophyll *a* to chlorophyll *b* is typically 1:0.33, but it can change towards an increased concentration of chlorophyll *b* due to adaptation to low light conditions [61]; the ratio can reach 6.2:1 [62]. This suggests that the content of chlorophyll *b* and the ratio of chlorophyll *a* to chlorophyll *b* may be a result of microalgae strain adaptation to different environmental conditions in their natural habitats. This explains the difference in the content and ratio of chlorophyll *a* and chlorophyll *b* in isolates of the same species grown under the same conditions. In particular, the ratio of chlorophyll *a* to chlorophyll *b* for *B. minor* MZ–Ch31 was 1:0.34, and for *B. minor* MZ–Ch39, it was 1:0.61.

In the literature, several scientists have claimed that the chlorophyll content of the genus *Bracteacoccus* varies greatly depending on ecological factors, the growth stage, and species differences between studied strains. Ratha et al. [45] provided the total content of chlorophyll *a*+*b* for the freshwater strain *Bracteacoccus* sp. MIC–G16, isolated from a mountain stream, and 2.5–110.0 mg/g DW after 18 days of cultivation, depending on the type of medium. This is 43.8 times and 116.5 times more than for *B. minor* MZ–Ch31 and *B. minor* MZ–Ch39 during early stationary growth. Minyuk et al. [38] investigated one of the authentic soil strains, *B. minor* ACKU 506-06 (=SAG 221-1). Its chlorophyll *a* content was 1.0–1.75 mg/g DW and 1.25 mg/L, 2.7 and 3 times lower than the *B. minor* MZ–Ch31 levels. Another strain, *B. minor* MZ–Ch39, had chlorophyll *a* concentration comparable to *B. minor* ACKU 506-06 (=SAG 221-1) [38].

The data obtained confirms that the chlorophyll content of strains of the same species isolated from different habitats may differ, despite the fact that they are cultivated in identical laboratory conditions. This likely results from individual adaptations that have developed in their native habitats.

### 3.3. Secondary Metabolites Content

Secondary metabolites are a group of low-molecular compounds that play various important roles in physiological processes. Tocopherols and carotenoids are the main fat-soluble antioxidants found in the membranes of chloroplasts, thylakoids, and other cellular structures [63]. Secondary metabolites have antioxidant properties: carotenoids [64,65,66], retinol [67], tocopherols [63,68]. Additionally, the antioxidant properties of the complex of low-molecular antioxidants are higher. For carotenoids, there is a synergistic effect of antioxidant properties when combined with polyphenols [69] and tocopherols [70,71,72,73]. The antioxidant activity of retinol and retinoids is significantly higher when α-tocopherol is present [67,74]. Considering the above, the complex interactions of carotenoids, retinol, and α-tocopherol with other antioxidants and pro-oxidants are specific to living organisms. This determines peroxidation processes in cells and the overall antioxidant capacity. This, in turn, determines differences in antioxidant resistance between strains, as observed in our study. In *B. minor* MZ–Ch31, antioxidant resistance is lower, against the background of increased concentrations of carotenoids and α-tocopherol, and a 16.9-fold decrease in retinol compared to *B. minor* MZ–Ch39. This is due to the higher antioxidant properties of retinol compared to carotenoids and α-tocopherol [67,74] as well as their synergistic interaction [70,71,72,73]. The antioxidant synergy of retinol–α-tocopherol is likely greater than that of carotenoids–α-tocopherol. In addition, it is worth considering the increased activity of AOS enzymes in *B. minor* MZ–Ch39 because enzymes have higher efficiency and rate of ROS inactivation.

As for the total content of secondary metabolites, there are significant differences in the content of carotenoids compared to other representatives of the genus *Bracteacoccus*. *B. minor* MZ–Ch31, with a carotenoid concentration of 6.80 mg/g DW and 9.18 mg/L, had a comparable or increased carotenoid content relative to other highly productive strains of *Bracteacoccus*. For example, Lukavský et al. [5] determined that the carotenoid content of snow epiphytic strain *B. bullatus* CCALA 1120 was 10.10 mg/L. The freshwater strain *B. aggregatus* BM5/15 contained carotenoids at 6.30 mg/g DW [40]. The freshwater strain *B. pseudominor* BERC09 had a carotenoid content of 2.0 mg/g DW, and with nitrogen starvation, this value reaches 8.40–11.90 mg/g DW [41]. Minyuk et al. [38] showed that the carotenoid content in the strain *B. minor* ACKU 506-06 (=SAG 221-1) was in the range of 6.30 mg/g DW at the logarithmic growth stage and 4.80 mg/g DW during stationary growth. The freshwater strain *Bracteacoccus* sp. MIC-G16 contained 16.0 mg/g DW carotenoids [45].

The total retinol content in Chlorophyta ranges from 10 to 4280 µg/g [2]. The retinol content in *B*. *minor* MZ–Ch 39 and *B*. *minor* MZ–Ch31 is at the lower end of the range.

According to Del Mondo et al. [2], the epiphytic strain *Bracteacoccus* sp. SAG 2137, isolated from the surface of spruce needles in Nerre-Risager, Denmark, contained 165.5 µg/g of α-tocopherol in the stationary growth phase. In contrast, *B*. *minor* MZ–Ch31 contained 73.4% more α-tocopherol than *Bracteacoccus* sp. SAG 2137. *B*. *minor* MZ–Ch31 is comparable to other highly productive strains. High concentrations of α-tocopherol make it a biotechnologically valuable producer of this metabolite. Our results for the strains *B. minor* MZ–Ch31 and *B. minor* MZ–Ch39, as well as the literature data, support the environmentally determined specificity of carotenoid and tocopherol accumulation. The results demonstrating ecologically determined changes in the content of α-tocopherol were also known for *Chlorella vulgaris* Beijerinck. Four strains of *Chlorella vulgaris* were isolated from two regions (Kedah and Terengganu) of Malaysia [27]. Under identical conditions of laboratory cultivation on BBM medium, the content of α-tocopherol in these strains varied in the range of 1250–3500 µg/g fresh weight. The high content of α-tocopherol in the biomass of *Chlorella vulgaris* isolated from the Terengganu region is the result of exposure to specific environmental factors common in the coastal waters of Terengganu, such as high ultraviolet radiation, fluctuations in salinity and temperature [27]. Accordingly, the high alpha-tocopherol content may be the result of adaptation to these effects.

### 3.4. Lipid Content

The lipid content among previously studied representatives of *Bracteacoccus* could reach 63%. Depending on the cultivation conditions, *B. minor* SAG 221-1 (=CCAP 221/1, =UTEX 66, =ACKU 506-06, =IBSS-88) contained lipids from 38 to 63%, with a medium containing 0.05 M sodium acetate [38]. *Bracteacoccus* sp. MIC-G16 contained 9.78–12.0% in various types of media, and *B. pseudominor* BERC09 reached 39.0–42.4% lipids under optimal lighting, temperature, and medium composition [41]. The strain *B. minor* MZ–Ch31 accumulates 31.4% of lipids when cultured on standard BBM medium, making it a promising strain for lipid production through stress-induced lipogenesis. At the same time, *B. minor* MZ–Ch39 has a lipid level comparable to the previously studied *Bracteacoccus* sp. MIC-G16 [45].

### 3.5. Antioxidant Activity Coefficient, Antioxidant Enzymes Activity, K_AAC_, and Lipid Peroxidation Substances

The coefficient of antioxidant activity (K_AAC_) is a comprehensive indicator of the status of the antioxidant system, including both enzymatic components and low-molecular antioxidants. Another characteristic of AOS is the content of secondary lipid degradation products. The main factor in the accumulation of secondary lipid degradation products is the overproduction of ROS, which the antioxidant defense system is unable to neutralize. TBARS and TBARS_in_ are products of LPO, specifically the degradation of unsaturated C18 fatty acids [75,76]. The low content of TBARS and TBARS_in_ in the cells of *B. minor* MZ–Ch39 explains the increased stability of the antioxidant defense system, and increased K_AAC_ confirms this. The low content of LPO products in *B. minor* MZ–Ch39 is due to the increase in CAT and GPx activity, as well as the high content of retinol compared to *B. minor* MZ–Ch31, and their synergistic effect combined with α-tocopherol also contributes to this [67,74]. Under stress, an increase in the content of LPO products is accompanied by a synchronous change in SOD activity in the microalgae biomass [25,77]. At the same time, CAT-activity is more specific, and an increase in CAT activity does not always accompany an increase in LPO products [25]. For exemple, these changes are synchronous for *Dunaliella salina* (Dunal) Teodoresco V-101 [77]. Earlier, in experiments with *Chlorococcum oleofaciens* Trainor et H.C. Bold CAMU MZ–Ch4, showed that CAT and SOD activities had an inverse correlation with the amount of α-tocopherol and retinol, and TBA-reactive substances (TBARS, TBARS_in_) had an inverse relationship with GPx activity [78].

The increased antioxidant activity of *B. minor* MZ–Ch39, with high activity of the AOS enzyme system, indicates its optimal functional state, in contrast to *B. minor* MZ–Ch31, where K_AAC_ and AOS enzyme activity are lower. It is worth noting that there was no significant difference in SOD activity between the two strains. This is due to the saturation of the *B. minor* MZ–Ch39 cell with hydrogen peroxide under conditions of SOD superoxide conversion [79]. The increased activity of antioxidant enzymes is associated with the saturation of their active centers by the substrate, under the condition of maintaining redox balance.

The decrease in GPx activity in *B. minor* MZ–Ch31 is associated with a low concentration of organic hydroperoxides. These hydroperoxides decompose into secondary LPO products [80], as confirmed by increased levels of TBARS and TBARS_in_ in dry biomass. In addition, GPx has a lower rate of substrate conversion compared to CAT and SOD. This is associated with the use of the GPx-specific coenzyme, glutathione, whose synthesis and regeneration are energy-consuming processes [81,82]. In *Chattonella* spp. biomass detected an increased role of GPx in protecting against oxidative damage with low CAT-activity [83].

Under these conditions, the functional activity of the AOS in *B. minor* MZ–Ch31 is lower, and it is mainly realized by low–molecular antioxidants, while in *B. minor* MZ–Ch39, it is realized by AOS enzymes. This is due to the generation of ROS as a result of the interaction of oxygen with photosynthetic pigments [84], which is logical considering the high content of chlorophyll in *B. minor* MZ–Ch31. ^1^O_2_ is the main electrophilic agent that affects UFA. The high content of LPO products confirms this. The main products that quench ^1^O_2_ radicals are carotenoids and tocopherols [84,85,86]. The content of these substances is higher in *B. minor* MZ–Ch31 than in *B. minor* MZ–Ch39.

The differences in the activity of antioxidant enzymes observed in the *B*. *minor* MZ–Ch31 and *B*. *minor* MZ–Ch39 may be the result of a complex set of factors. It is known that different species and strains may have varying antioxidant capacities due to their genetic characteristics [87]. It is also known that acclimatization to stress increases the content of low molecular weight antioxidants and the activity of antioxidant enzymes [22,26]. The strains MZ–Ch31 and MZ–Ch39 were isolated from habitats with specific environmental factors. The habitats of hardwood and coniferous plantations differed in the amount of light on the soil surface, the soil had different pH levels, and the amount of nutrients and other indicators. Thus, the acclimatization of the *B. minor* MZ–Ch31 and MZ–Ch39 strains proceeded differently, which affected their antioxidant response.

Previously reported about the species-specificity of the antioxidant profiles of microalgae, as well as their dependence on the habitat [27]. Using the example of four strains of *Chlorella vulgaris* and two strains of *Nannochloropsis oceanica* Suda et Miyashita isolated from various biotopes in Malaysia, they demonstrated that CAT and SOD activity within strains of the same species showed high variability. The ratio of the minimum and maximum CAT activity values for *Nannochloropsis oceanica* was 500, and for *Chlorella vulgaris*, was 175. SOD activity was less variable: for *Nannochloropsis oceanica*, the difference between the minimum and maximum values was 30%, while for *Chlorella vulgaris*, it was 3.1 times. Moreover, the reduced variability of SOD activity relative to CAT is comparable to the data for MZ–Ch31 and MZ–Ch39. Such differences noted that the variability of the studied enzymes activity was higher for marine *Nannochloropsis oceanica* and freshwater *Chlorella vulgaris* than for soil *B*. *minor*.

### 3.6. Fatty Acids Profile and Fatty Acids Unsaturation Index

Fatty acids play several vital roles in cells, including structural, energetic, and regulatory functions. They also serve as markers of cell metabolic state [88,89]. The ratio of saturated and unsaturated fatty acids in the composition of cellular structures plays a crucial role in the adaptive function of cells and determines their resistance to ROS. This ratio allows us to assess the functional state of cells and the nature of their protective functions by analyzing the fatty acid composition.

As previously mentioned, the FA profiles of *B. minor* MZ–Ch31 and *B. minor* MZ–Ch39 differ quite significantly in both their quantitative and qualitative composition. The reason for this is due to environmental factors, specifically, the parameters of the habitat from which the strains were isolated. The literature data confirms this. Using the example of three strains of *B. bullatus* isolated from different habitats: *B. bullatus* MZ–Ch11 (soil, Acacia Forest) [39], *B. bullatus* CCALA 1120 (snow, Sierra Nevada Mountain range) [5], and *B. bullatus* MZ–Ch32 (Staro-Berdyansk forest litter) [36]. Data demonstrate a wide range of fatty acid profile types. For *B. bullatus* MZ–Ch32, the absence of stearic 18:0 and hexadienoic 16:2 fatty acids and α-linolenic acid 18:3n-3 is specific, with a maximum content of linoleic acid 18:2n-6 at 23.8%, compared to *B. bullatus* MZ–Ch11 and *B. bullatus* CCALA 1120. The strain *B. bullatus* MZ–Ch11 had a content of 63.8% oleic acid 18:1, while *B. bullatus* CCALA 1120 had 22.6%, and *B. bullatus* MZ–Ch32 had 43.2%. *B. bullatus* CCALA 1120 had an increased content of α-linolenic acid 18:3n-3 at 17.4% compared to *B. bullatus* MZ–Ch11, which had 0.27%, and *B. bullatus* MZ–Ch32, which had 0%. If we consider these changes from the perspective of species affiliation, the impact of this factor is less significant. The fatty acid profile of freshwater *B. bullatus* BM5/15 and *B. bullatus* CCALA 1120, isolated from different environments, such as freshwater [40] and snow surfaces [5], had greater similarity in 18:0, 18:1, 18:2, and 18:3 content compared to representatives of the same species. Our study confirms this using the example of *B. minor* strains.

From the point of view of the overall content of individual fatty acids, it is worth noting that *B. minor* MZ–Ch39 has the highest content of linoleic acid 18:2n-6 at 27.1%. This value is higher than the content of C18:2 in *B. minor* MZ–Ch39 (15.6%) and in the previously studied strain *B. bullatus* MZ–Ch11 (13.9%). The linoleic acid 18:2n-6 content in other *Bracteacoccus* species was as follows: *B. bullatus* CCALA 1120 18.3%, *B. bullatus* MZ–Ch32 23.8%, and *B. aggregatus* BM5/15 18.2%. At the same time, strain *B. minor* had the highest amount of α-linolenic 18:3n-3 (31%) among all strains described above (0–17.4%).

Comparing strains to *B. minor* MZ–Ch31 and *B. minor* MZ–Ch39, based on the distribution of the mass fraction of fatty acids in the total pool, we found that both strains actively metabolize C18 fatty acids. The conversion proceeds without further β-oxidation of 18-carbon FA in peroxisomes, since the total content of this type of metabolite for *B. minor* MZ–Ch31 and *B. minor* MZ–Ch39 is comparable (63.6% and 63.1%, respectively, of the total amount of FA). This may be one of the systemic features. There was no significant difference in the degree of fatty acid unsaturation index between the strains, even though polyunsaturated fatty acids are the main substrate for LPO [17,76,90]. This indicates that the mechanism of lowering total UI is secondary to the formation of an antioxidant response. This is a main argument for using strains *B. minor* MZ–Ch31 and *B. minor* MZ–Ch39 for stress-induced production of essential n-3 and n-6 fatty acids. In this context, it is worth considering that PUFA is the main substrate for LPO [76]. Accordingly, *B. minor* MZ–Ch31 has fewer LPO-resistant lipids, since α-linolenic acid 18:3n-3, whose content is 2 times higher than in *B. minor* MZ–Ch39, mainly forms fatty acid UI.

The data obtained, combined with data from other researchers, confirm that differences in the fatty acid composition of strains from the same species originating from different biotopes are clearly the result of individual adaptations to specific environmental conditions. These adaptations are fixed at the genetic level and are evident even when the strains are cultivated in laboratory settings.

## 4. Materials and Methods

### 4.1. Microalgae Strains

The novel strains MZ–Ch31 and MZ–Ch39 were isolated by micropipetting from algae enrichment cultures using an inverted Olympus CKX53 microscope (Olympus, Japan). Small amounts of soil samples were taken to obtain enrichment cultures, placed in Petri dishes, and moisturized. The strain MZ–Ch31 was isolated from the soil of an urban park with a *Robinia pseudoacacia* plantation with sparse grass coverage and an underdeveloped forest litter (Melitopol, Russia). The strain MZ–Ch39 was isolated from a pine forest with well-developed undergrowth and developed forest litter (Ramon, Voronezh region, Russia). The soil strains MZ–Ch31 and MZ–Ch39 were deposited in the Culture and Barcode Collection of Microalgae and Cyanobacteria ‘Algabank’ (WDCM 1318) at К.А. Timiryazev Institute of Plant Physiology RAS (Moscow, Russia) and the Collection of Algae at Melitopol State University CAMU (WDCM 1158) as perpetually transferred pure cultures.

Light microscopic observations were performed with a Zeiss Axio Scope A1 (Carl Zeiss Microscopy GmbH, Göttingen, Germany) microscope equipped with an oil immersion objective (Plan-apochromatic ×100/n.a.1.4, Nomarski differential interference contrast, DIC) and a Zeiss Axio Cam ERc 5s camera (Carl Zeiss NTS Ltd., Oberkochen, Germany). Observation of the strain lasted from 24 h to 6 months. The culture was maintained on the BBM medium [91].

As reactors, we used flat-bottomed flasks with a volume of 250 mL with sealed lids and a system to ensure the consistency of the composition of the gas-air mixture in the flask. The Hailea ACO-308 aquarium compressor (Guangdong Hailea Group Co., Ltd., Raoping County, Guangdong Province, China) supplied air for aeration of the cell culture with air. The air went through a glass tube with an internal diameter of 4 mm at a speed of 0.1 L/min. To prevent bacterial contamination of the culture, we used a bacterial ventilation filter (GSV, Italy) with a diameter of 40 mm (pore size 0.22 µm), the filter was in the gap between the compressor and the glass tube. This made it possible to maintain the culture in an algologically pure state. To assess the growth and biochemical characteristics of the strain, it was grown in Erlenmeyer flasks with a volume of 250.0 mL and 150.0 mL of BBM at 23.0 ± 2.0 °C. The initial cell concentration was 2.89 × 10^5^ cells/mL. Cell concentrations were measured with a C100 Automated Cell Counter (RWD Life Science, China). The light intensity was 5000 lx (70.0 µmol photons/s × m^2^) with a color temperature of 4000 K, and the illumination mode was 16:8 (light:dark). These lighting conditions are standard. The light intensity and color temperature were measured using the Hopoocolor OHSP–350P (Hangzhou Hopoo Light and Color Technology Co., Ltd., Hangzhou, China). Cell cultures were cultured with constant shaking at a frequency of 60 rpm by KJ-201 BD (Pioway Medical Lab Equipment, China). The cultivation process lasted 21 days.

### 4.2. Molecular Analysis

Molecular analysis, performed herein, was carried out according to the algorithm, performed in Maltsev et al. [36,92]. The DNA of the investigated strains MZ–Ch31 and MZ–Ch39 was extracted using Chelex 100 Chelating Resin, molecular biology grade (Bio-Rad Laboratories, Hercules, CA, USA), according to the manufacturer’s protocol 2.2. The V4 barcoding region of the 18S rDNA nuclear gene with a length of 484–485 bp was amplified using the primer pair D512for and D978rev from Zimmermann et al. [93] and the following reaction conditions: 95 °C for 5 min (initial denaturation), 35 cycles consisting of 95 °C for 30 s (denaturation), 52 °C for 40 s (annealing), 72 °C for 50 s (elongation), and a final extension step at 72 °C for 5 min. Amplification of the ITS1–5.8S rDNA–ITS2 region with a length of 548–550 bp was carried out using the ITS1 and ITS4 primer pair [94]; reaction conditions included 95 °C for 5 min (initial denaturation), 45 cycles consisting of 94 °C for 30 s (denaturation), 60 °C for 30 s (annealing), 72 °C for 60 s (elongation), and a final extension step at 72 °C for 5 min.

Amplifications were performed using premade polymerase chain reaction (PCR) mastermixes (ScreenMix by Evrogen, Moscow, Russia). The PCR products were visualized by horizontal electrophoresis in 1.0% agarose gel stained with SYBR^TM^ Safe (Life Technologies, Carlsbad, CA, USA). The products were purified with a mixture of FastAP, 10× FastAP Buffer, Exonuclease I (Thermo Fisher Scientific, Waltham, MA, USA), and water. The sequencing was performed using a Genetic Analyzer 3500 instrument (Applied Biosystems, Waltham, MA, USA).

Editing and assembling of the consensus sequences were carried out by processing the direct and reverse chromatograms in Ridom TraceEdit ver. 1.1.0 (Ridom GmbH, Münster, Germany) and Mega ver. 7.0.26 software [95]. The 18S rDNA and the ITS1–5.8S rDNA–ITS2 sequences of the novel strains were included in the alignments of 27 representatives of Sphaeropleales (Bracteococcaceae and Pseudomuriellaceae clades) from GenBank (taxa names and accession numbers are given in Figure 2). Nucleotide sequences of *Ankyra judayi* (G.M. Smith) Fott and *Sphaeroplea robusta* Buchheim et Hoffman (Sphaeropleaceae clade) were used as the external group. The 18S rDNA and ITS1–5.8S rRNA–ITS2 sequences of all strains (including outgroups) were aligned in Mega ver. 7.0.26 software according to their secondary structure. The resulting alignments had lengths of 1103 characters.

The data set was analyzed using the BI method implemented in Beast ver. 1.10.1 software (BEAST Developers, Auckland, New Zealand) [96] to construct a phylogeny. The most appropriate substitution model for the alignment partition and shape parameter α were estimated using the Bayesian information criterion (BIC) as implemented in jModelTest ver. 2.1.10 (Vigo, Spain) [97]. This BIC-based model selection procedure selected the K80 model for the 18S rRNA gene and TrNef+G with α = 0.4050 for the ITS1–5.8S rDNA–ITS2 region. We used the HKY model of nucleotide substitution instead of TrNef, given that it was the best matching model available for BI. A Yule process tree prior was used as a speciation model. The analysis ran for 5 million generations with chain sampling every 1,000 generations. The parameter-estimated convergence, effective sample size (ESS), and burn-in period were checked using the Tracer ver. 1.7.1 software (MCMC Trace Analysis Tool, Edinburgh, UK) [96]. The initial 25% of the trees were removed, and the rest were retained to reconstruct a final phylogeny. The phylogenetic tree and posterior probabilities of its branching were obtained based on the remaining trees, having stable estimates of the parameter models of nucleotide substitutions and likelihood. The ML analysis was performed using RA×ML software [98]. The nonparametric bootstrap analysis with 1000 replicas was used. FigTree ver. 1.4.4 (University of Edinburgh, Edinburgh, UK) and Adobe Photoshop CC ver. 19.0 software (Adobe, San Jose, CA, USA) was used for viewing and editing the trees.

### 4.3. α-Tocopherol and Retinol Content Measurement

To prepare a sample of 30 mg of microalgae biomass, it was previously saponified at 80–85 °C in a KOH solution in ethanol with the addition of 20 mg of ascorbic acid (an antioxidant). α-Tocopherol was isolated from the resulting hydrolysate by sequential extraction with diethyl ether in volumes of 2, 1, and 1 mL. The essential extract was washed with water until a neutral reaction on a universal indicator paper. Next, the extract was dried by freezing at -18 °C, and filtered through a membrane filter with a pore size of 0.22 µm. The resulting extract was evaporated in a vacuum at 45–55 °C and dissolved in 1 mL of methanol for analysis. The volume of the injected sample was 10 µL.

Reverse-phase HPLC determined the content of a-tocopherol in biomass. Determination of α-tocopherol was carried out on a Chromatron-1411 liquid chromatograph (JSC Labtech, Russia) with a spectrophotometric UV/VID detector. α-Tocopherol detection was performed at a wavelength of 292 nm. An Inspire C18 column (5 µm 150 × 4.6 mm) was used as a carrier of the stationary phase. A mixture of methanol/water was used as the mobile phase in the ratio 96:4 (*v*:*v*), containing 2.5 mM acetic acid/sodium acetate [99]. The mixture was supplied at a rate of 1 mL/min. The separation time was 20 min. During separation, the column temperature was 30 °C. The retention time of α-tocopherol was determined by the retention time of a standard sample from a Merck kit (Merck KGaA, Darmstadt, Germany). For the quantitative determination of α-tocopherol, the calibration curve method was used, which was constructed based on the height and area of peaks obtained by chromatography of calibration solutions with a final concentration of 10–100 µg/mL. Calibration solutions were prepared by diluting a standard solution obtained by diluting α-tocopherol from a Merck kit (Merck KGaA, Darmstadt, Germany) in methanol, with an initial concentration of 1 mg/mL. The concentration was expressed in µg per g of dry weight.

The retinol content was determined by thin-layer chromatography. Thin-layer chromatography plates TLC 60 F254 silica gel (Merck KGaA, Darmstadt, Germany) were used as the carrier of the stationary phase. A mixture of n-hexane-diethyl ether in a 9:1 ratio by volume was used as the mobile phase [100]. After chromatography, the chromatographic plate was immersed for 5 s in a 1% solution of phosphoric acid in ethanol, followed by heating to 110–120 °C in a stream of hot air. After heating, retinol appears as blue spots on a yellow background. The location of the retinol spots on the plate was determined by comparison with the Rf of a standard retinol solution (Sigma-Aldrich, St. Louis, MI, USA). The yellow background was removed by exposure in a chamber saturated with ammonia vapor. To calculate the retinol content, the plates were scanned using a Canon MF 3010 scanner and processed using the Sorbfil TLC Videodensitometer ver. 2.3.0 software. The height and area of the peaks corresponding to the retinol spots on the plate were compared with the data of a calibration graph constructed using retinol calibration solutions (Sigma-Aldrich, St. Louis, MI, USA) with a final concentration of 10–100 µg/mL. The concentration was expressed in µg/g DW.

### 4.4. Antioxidant Enzyme Activity Measurement

To analyze the enzyme activity, we previously prepared an enzyme extract. To do this, 0.1 g of biomass was separated from the medium by centrifugation (10 min at 3000 rpm), then 0.9 mL of phosphate buffer (0.01 M; pH = 7.4) was added to the biomass and homogenized for 30 s using a JY92-IIN ultrasonic homogenizer (Scientz Biotechnology, China) (horn diameter 6 mm, power 65 W, frequency 25 kHz) at a constant temperature of 2–4 °C. The resulting homogenate was centrifuged for 10 min at 10,000 rpm, and the resulting supernatant was used to analyze enzyme activity on the same day.

The CAT activity (EC 1.11.1.6) was determined using the Hamza and Hadwan [101] method with modification. The volume of the reaction medium was 3 mL. To start the reaction, 0.05 mL of the supernatant was added to 2 mL of hydrogen peroxide (10 mM) and incubated for 10 min at 30 °C. After 10 min, 1 mL of the working reagent (0.04 M aniline sulfate, 0.125 M hydroquinone, 0.04 M ammonium molybdate) was added to the medium, kept at room temperature for 10 min, and the spectrophotometric measurement was taken at 550 nm. The concentration of reacted hydrogen peroxide to express activity was determined by the calibration curve of the interaction of hydrogen peroxide of known concentration with the working solution.

GPx activity (EC 1.11.1.9) has been determined by the Sattar et al. [102] method with modification. The reaction medium contained 1.5 mL of phosphate buffer (0.01 M; pH = 7.4), 0.2 mL of reduced glutathione (2 mM), and 0.05 mL of a supernatant. The reaction was started by adding 0.2 mL of hydrogen peroxide (2.1 mM) and incubating for 60 min. After that, we added 3 mL of sodium hydrophosphate and 1 mL of Elman’s reagent, and we kept the samples for 10 min at 37 °C. The OD of the solution was measured at 412 nm. To calculate the concentration of reacted glutathione and express enzyme activity, the molar extinction coefficient of the glutathione complex with Elman reagent was used [102].

SOD activity (EC 1.15.1.1) was determined using the Sirota [103] method. To determine the enzyme activity, we prepared a reaction mixture that contained 2 mL of carbonate buffer (0.2 M; pH = 10.5), nitroblue tetrazolium (50 µM), and 0.05 mL of a supernatant. The reaction was started by adding epinephrine chloride (0.23 mM) to the incubation medium and incubating for 3 min at 25 °C. The OD was measured at 560 nm before and 3 min after adding adrenaline chloride. The activity was calculated using the equation by Sirota [103] method.

### 4.5. Other Biochemical Parameters

Fatty acid profile analysis by gas chromatography was performed according to Maltseva et al. [104]. Chlorophylls *a*,*b*, carotenoids, lipids, protein content, and TBARS and TBARS_in_ were performed according to Maltseva et al. [78], as well as the calculation of the coefficient of antioxidant activity (K_AAC_) [20]. The fatty acid UI was also calculated—the total equivalent concentration of fatty acids in mmol/g relative to the number of double bonds, which can act as an indicator of the resistance of lipids of biological membranes to peroxide oxidation. The calculation was carried out according to Kaszycki et al. [105]. Additionally, the molar masses of FA were used. The UI was expressed in mmol/g of FA.

### 4.6. Data Analysis

Statistics obtained in Microsoft Excel ver. 1903 (Microsoft Corporation, Redmond, Washington, USA) software using single-factor dispersion analysis (ANOVA). The reliability of the differences between the indicators was calculated using the Tukey–Kramer posterior test. The differences at *p* ≤ 0.05 were considered reliable.

## 5. Conclusions

In this study, the accumulation of metabolites and the antioxidant response of *Bracteacoccus* strains collected in soils of various forest ecosystems were successfully analyzed.

It was found that *B*. *minor* MZ–Ch39 had a higher antioxidant activity compared to *B*. *minor* MZ–Ch31. The content of TBARS and TBARS_in_ in the *B*. *minor* MZ–Ch39 cells was lower than that of MZ–Ch31. The antioxidant response in *B*. *minor* MZ–Ch39 was realized by high CAT, GPx activity, and accumulation of retinol. In *B*. *minor* MZ–Ch31, the antioxidant response was associated with the accumulation of α-tocopherol and carotenoids. The strains did not differ in terms of SOD activity.

We suggest that differences in the formation of the antioxidant response may be related to the acclimatization of these strains to specific environmental factors of natural biotopes. The established differences in the content of lipids, the profile of fatty acids, pigments, and the rate of biomass accumulation in the strains demonstrate their ecological determinism.

From a biotechnological point of view, *B*. *minor* MZ–Ch31 biomass is a valuable source of lipids rich in omega-3 fatty acids, α-tocopherol, and carotenoids. *B*. *minor* MZ–Ch39 has the potential to produce lipids enriched with essential omega-6 fatty acids.

## Figures and Tables

**Table 1:** Fatty acid profile of strains *B. minor* MZ–Ch31, *B. minor* MZ–Ch39 (BBM medium; culture volume 150 mL; cultivation time 21 days, M ± SD, n = 3).

FA Code	FA Name	MZ–Ch31		MZ–Ch39	
		ω, %	UI, mmol/g	ω, %	UI, mmol/g
14:0	Myristic acid	0	-	0.25 ± 0.01*	-
16:0	Palmitic acid	22.78 ± 1.14	-	19.53 ± 0.98	-
18:0	Stearic acid	13.03 ± 0.65	-	7.61 ± 0.38*	-
24:0	Lignoceric acid	0.61 ± 0.03	-	0*	-
26:0	Cerotic acid	0.53 ± 0.03	-	0*	-
16:1n-7	Palmitoleic acid	1.45 ± 0.07	5.71	1.34 ± 0.07	5.28
18:1n-9	Oleic acid	3.95 ± 0.20	14.01	12.24 ± 0.61 *	43.40
16:2n-6	Hexadecadienoic acid	2.95 ± 0.15	23.41	7.19 ± 0.36 *	57.06
18:2n-6	Linoleic acid	15.59 ± 0.78	111.36	27.06 ± 1.35 *	193.29
16:3n-3	Hexadecatrienoic acid	8.07 ± 0.40	96.84	8.58 ± 0.43	102.96
18:3n-3	α-Linolenic acid	31.04 ± 1.55	334.96	16.20 ± 0.81 *	174.82
total SFA		36.95		27.39	
total MUFA		5.40		13.58	
total PUFA		57.65		59.03	
total omega-3		39.11		24.78	
total omega-6		18.54		34.25	
ω3/ω6		2.11		0.72	
Total UI			586.29		576.81

Note. UI—FA unsaturation index, mmol/g; *—the differences are significant relative to *B. minor* MZ–Ch31 at the level *p* ≤ 0.05.

## Data Availability

The original contributions presented in this study are included in the article. Further inquiries can be directed to the corresponding authors.

## References

[1] Sathasivam R., Radhakrishnan R., Hashem A., Abd_Allah E.F. (2019). Microalgae metabolites: A rich source for food and medicine. Saudi J. Biol. Sci..

[2] Del Mondo A., Smerilli A., Sané E., Sansone C., Brunet C. (2020). Challenging microalgal vitamins for human health. Microb. Cell Fact..

[3] Gohara-Beirigo A.K., Matsudo M.C., Cezare-Gomes E.A., Carvalho J.C.M.D., Danesi E.D.G. (2022). Microalgae trends toward functional staple food incorporation: Sustainable alternative for human health improvement. Trends Food Sci. Technol..

[4] Katayama T., Rahman N.A., Khatoon H., Kasan N.A., Nagao N., Yamada Y., Takahashi K., Furuya K., Wahid M.E.A., Yusoff F.M. (2022). Bioprospecting of Tropical Microalgae for High-Value Products: N-3 Polyunsaturated Fatty Acids and Carotenoids. Aquac. Rep..

[5] Lukavský J., Kopecký J., Kubáč D., Kvíderová J., Procházková L., Řezanka T. (2023). The alga *Bracteacoccus bullatus* (Chlorophyceae) isolated from snow, as a source of oil comprising essential unsaturated fatty acids and carotenoids. J. Appl. Phycol..

[6] Aslam A., Rasul S., Bahadar A., Hossain N., Saleem M., Hussain S., Rasool L., Manzoor H. (2021). Effect of Micronutrient and Hormone on Microalgae Growth Assessment for Biofuel Feedstock. Sustainability.

[7] Satpati G.G., Pal R. (2021). Co-cultivation of *Leptolyngbya tenuis* (Cyanobacteria) and *Chlorella ellipsoidea* (green alga) for biodiesel production, carbon sequestration, and cadmium accumulation. Cur. Microbiol..

[8] Maltsev Y., Kulikovskiy M., Maltseva S. (2023). Nitrogen and phosphorus stress as a tool to induce lipid production in microalgae. Microb. Cell Fact..

[9] Vijayan J., Alvarez S., Naldrett M.J., Maliva A., Wase N., Riekhof W.R. (2024). Nitrogen starvation leads to TOR kinase-mediated downregulation of fatty acid synthesis in the algae *Chlorella sorokiniana* and *Chlamydomonas reinhardtii*. BMC Plant Biol..

[10] Tripathi B.N., Mehta S.K., Amar A., Gaur J.P. (2006). Oxidative stress in *Scenedesmus* sp. during short- and long-term exposure to Cu^2+^ and Zn^2+^. Chemosphere.

[11] Pereira P., De Pablo H., Rosa-Santos F., Pacheco M., Vale C. (2009). Metal accumulation and oxidative stress in *Ulva* sp. substantiated by response integration into a general stress index. Aquat. Toxicol..

[12] Melegari S.P., Perreault F., Moukha S., Popovic R., Creppy E.E., Matias W.G. (2012). Induction to oxidative stress by saxitoxin investigated through lipid peroxidation in Neuro 2A cells and *Chlamydomonas reinhardtii* alga. Chemosphere.

[13] Kováčik J., Klejdus B., Babula P. (2014). Oxidative stress, uptake and bioconversion of 5-fluorouracil in algae. Chemosphere.

[14] Almeida A.C., Gomes T., Langford K., Thomas K.V., Tollefsen K.E. (2017). Oxidative stress in the algae *Chlamydomonas reinhardtii* exposed to biocides. Aquat. Toxicol..

[15] Mailloux R.J., Singh R., Brewer G., Auger C., Lemire J., Appanna V.D. (2009). α-Ketoglutarate dehydrogenase and glutamate dehydrogenase work in tandem to modulate the antioxidant α-ketoglutarate during oxidative stress in *Pseudomonas fluorescens*. J. Bacteriol..

[16] McLain A.L., Szweda P.A., Szweda L.I. (2011). α-Ketoglutarate dehydrogenase: A mitochondrial redox sensor. Free Radical Res..

[17] Naudí A., Jové M., Ayala V., Portero-Otín M., Barja G., Pamplona R. (2013). Membrane lipid unsaturation as physiological adaptation to animal longevity. Front. Physiol..

[18] Kurutas E.B. (2015). The importance of antioxidants which play the role in cellular response against oxidative/nitrosative stress: Current state. Nutr. J..

[19] Cao Y., Yang K., Liu W., Feng G., Peng Y., Li Z. (2022). Adaptive responses of common and hybrid bermudagrasses to shade stress associated with changes in morphology, photosynthesis, and secondary metabolites. Front. Plant Sci..

[20] Yakoviichuk A., Krivova Z., Maltseva S., Kochubey A., Kulikovskiy M., Maltsev Y. (2023). Antioxidant Status and Biotechnological Potential of New *Vischeria vischeri* (Eustigmatophyceae) Soil Strains in Enrichment Cultures. Antioxidants.

[21] Chakdar H., Hasan M., Pabbi S., Nevalainen H., Shukla P. (2021). High-throughput proteomics and metabolomic studies guide re-engineering of metabolic pathways in eukaryotic microalgae: A review. Bioresour. Technol..

[22] Nowicka B. (2022). Heavy metal-induced stress in eukaryotic algae—Mechanisms of heavy metal toxicity and tolerance with particular emphasis on oxidative stress in exposed cells and the role of antioxidant response. Environ. Sci. Pollut. Res..

[23] Chokshi K., Pancha I., Ghosh A., Mishra S. (2017). Nitrogen starvation-induced cellular crosstalk of ROS-scavenging antioxidants and phytohormone enhanced the biofuel potential of green microalga *Acutodesmus dimorphus*. Biotechnol. Biofuels.

[24] Nong Q.Y., Liu Y.A., Qin L.T., Liu M., Mo L.Y., Liang Y.P., Zeng H.H. (2021). Toxic mechanism of three azole fungicides and their mixture to green alga *Chlorella pyrenoidosa*. Chemosphere.

[25] Qiu C., Wang W., Zhang Y., Zhou G.-J., Bi Y. (2022). Response of Antioxidant Enzyme Activities of the Green Microalga *Chlorococcum* sp. AZHB to Cu^2^+ and Cd^2+^ Stress. Sustainability.

[26] Mishra Y., Bhargava P., Thapar R., Srivastava A.K., Rai L.C. (2008). A comparative study of antioxidative defense system in the copper and temperature acclimated strains of *Anabaena doliolum*. World J. Microbiol. Biotechnol..

[27] Awang N.A., Jusoh M., Said N.F., Yusuf N., Lani M.N., Ahmad F.T. (2025). Microalgae as potential antioxidants: Assessment of antioxidant capacities in microalgae from selected regions of Peninsular Malaysia. Pertanika J. Trop. Agric. Sci..

[28] Maltsev Y., Krivova Z., Maltseva S., Maltseva K., Gorshkova E., Kulikovskiy M. (2021). Lipid accumulation by *Coelastrella multistriata* (Scenedesmaceae, Sphaeropleales) during nitrogen and phosphorus starvation. Sci. Rep..

[29] Nayana K., Sudhakar M.P., Arunkumar K. (2022). Biorefinery potential of *Coelastrella* biomass for fuel and bioproducts—A review. Biomass Conv. Bioref..

[30] Trivedi J., Aila M., Bangwal D.P., Kaul S., Garg M.O. (2015). Algae based biorefinery—How to make sense?. Renew. Sustain. Energy Rev..

[31] Piñeros R.J., Manrique Ruíz I.G., Herrera J.A., Fernández D. (2019). Evaluación de carotenoides y lípidos en la microalga *Scenedesmus dimorphus* a escala laboratorio. Revista Mutis.

[32] Hariram V., John J.G., Sangeethkumar E., Gajalakshmi B., Ramanathan V. (2022). *Scenedesmus obliquus* and *Chlorella vulgaris*—A prospective algal fuel source. Nat. Environ. Poll. Tech..

[33] Yu X., Zhao P., He C., Li J., Tang X., Zhou J., Huang Z. (2012). Isolation of a novel strain of *Monoraphidium* sp. and characterization of its potential application as biodiesel feedstock. Bioresour. Technol..

[34] Georgiou D., Exarhopoulos S., Charisis A., Simitsis S., Papapanagiotou G., Samara C., Katsiapi M., Kountrias G., Bouras S., Katsoulas N. (2024). Valorization of *Monoraphidium* sp. microalgal biomass for human nutrition applications. J. Appl. Phycol..

[35] Maltsev Y., Maltseva I., Maltseva S., Kociolek J.P., Kulikovskiy M. (2021). A new species of freshwater algae *Nephrochlamys yushanlensis* sp. nov. (Selenastraceae, Sphaeropleales) and its lipid accumulation during nitrogen and phosphorus starvation. J. Phycol..

[36] Maltsev Y.I., Maltseva I.A., Maltseva S.Y., Kulikovskiy M.S. (2020). Biotechnological potential of a new strain of *Bracteacoccus bullatus* (Sphaeropleales, Chlorophyta) as a promising producer of omega-6 polyunsaturated fatty acids. Russ. J. Plant Physiol..

[37] Gatenby C.M., Orcutt D.M., Kreeger D.A., Parker B.C., Jones V.A., Neves R.J. (2003). Biochemical composition of three algal species proposed as food for captive freshwater mussels. J. Appl. Phycol..

[38] Minyuk G.S., Chelebieva E.S., Chubchikova I.N. (2014). Secondary carotenogenesis of the green microalga *Bracteacoccus minor* (Chodat) Petrova (Chlorophyta) in a two-stage culture. Inter. J. Algae.

[39] Mamaeva A., Namsaraev Z., Maltsev Y., Gusev E., Kulikovskiy M., Petrushkina M., Filimonova A., Sorokin B., Zotko N., Vinokurov V. (2018). Simultaneous increase in cellular content and volumetric concentration of lipids in *Bracteacoccus bullatus* cultivated at reduced nitrogen and phosphorus concentrations. J. Appl. Phycol..

[40] Chekanov K., Litvinov D., Fedorenko T., Chivkunova O., Lobakova E. (2021). Combined Production of Astaxanthin and β-Carotene in a New Strain of the Microalga *Bracteacoccus aggregatus* BM5/15 (IPPAS C-2045) Cultivated in Photobioreactor. Biology.

[41] Malik S., Ashraf M.U.F., Shahid A., Javed M.R., Khan A.Z., Usman M., Manivannan A., Mehmood M.A., Ashraf G.A. (2022). Characterization of a newly isolated self-flocculating microalga *Bracteacoccus pseudominor* BERC09 and its evaluation as a candidate for a multiproduct algal biorefinery. Chemosphere.

[42] Czerwik-Marcinkowska J., Mrozińska T. (2011). Epilithic algae from caves of the Krakowsko-Częstochowska Upland (Southern Poland). Acta Soc. Bot. Pol..

[43] Chekanov K., Fedorenko T., Kublanovskaya A., Litvinov D., Lobakova E. (2020). Diversity of carotenogenic microalgae in the White Sea polar region. FEMS Microbiol. Ecol..

[44] Chubchikova I.N., Drobetskaya I.V., Minyuk G.S., Dantsyuk N.V., Chelebieva E.S. (2011). Screening of unicellular green algae as a potential source of natural ketocaratenoids. 2. The growth and secondary carotenogenesis in some species of genus *Bracteacoccus* (Chlorophyceae). Mor. Ecol. J..

[45] Ratha S.K., Babu S., Renuka N., Prasanna R., Prasad R.B.N., Saxena A.K. (2013). Exploring nutritional modes of cultivation for enhancing lipid accumulation in microalgae. J. Basic Microbiol..

[46] Chekanov K., Shibzukhova K., Lobakova E., Solovchenko A. (2022). Differential Responses to UV-A Stress Recorded in Carotenogenic Microalgae *Haematococcus rubicundus*, *Bracteacoccus aggregatus*, and *Deasonia* sp.. Plants.

[47] Chekanov K. (2023). Diversity and Distribution of Carotenogenic Algae in Europe: A Review. Mar. Drugs.

[48] Santhakumaran P., Ayyappan S., Ray J.G. (2020). Nutraceutical applications of twenty-five species of rapid-growing green-microalgae as indicated by their antibacterial, antioxidant and mineral content. Algal Res..

[49] Goiris K., Muylaert K., Fraeye I., Foubert I., De Brabanter J., De Cooman L. (2012). Antioxidant potential of microalgae in relation to their phenolic and carotenoid content. J. Appl. Phycol..

[50] Safafar H., Van Wagenen J., Møller P., Jacobsen C. (2015). Carotenoids, Phenolic Compounds and Tocopherols Contribute to the Antioxidative Properties of Some Microalgae Species Grown on Industrial Wastewater. Mar. Drugs.

[51] Maltseva S.Y., Kulikovskiy M.S., Maltsev Y.I. (2022). Functional state of *Coelastrella multistriata* (Sphaeropleales, Chlorophyta) in an enrichment culture. Microbiology.

[52] Fučíková K., Flechtner V.R., Lewis L.A. (2012). Revision of the genus *Bracteacoccus* Tereg (Chlorophyceae, Chlorophyta) based on a phylogenetic approach. Nova Hedwigia.

[53] Koksharova O.A., Safronov N.A. (2022). The effects of secondary bacterial metabolites on photosynthesis in microalgae cells. Biophys. Rev..

[54] Chukhutsina V.U., Fristedt R., Morosinotto T., Croce R. (2017). Photoprotection strategies of the alga *Nannochloropsis gaditana*. Biochim. Biophys. Acta Bioenerg..

[55] Ptushenko V.V., Zhigalova T.V., Avercheva O.V., Tikhonov A.N. (2019). Three phases of energy-dependent induction of P^+^_700_ and Chl *a* fluorescence in *Tradescantia fluminensis* leaves. Photosynth. Res..

[56] Messant M., Krieger-Liszkay A., Shimakawa G. (2021). Dynamic changes in protein-membrane association for regulating photosynthetic electron transport. Cells.

[57] Gomez-Casati D.F., Barchiesi J., Busi M.V. (2022). Mitochondria and chloroplasts function in microalgae energy production. PeerJ.

[58] Sunil B., Talla S.K., Aswani V., Raghavendra A.S. (2013). Optimization of photosynthesis by multiple metabolic pathways involving interorganelle interactions: Resource sharing and ROS maintenance as the bases. Photosynth. Res..

[59] Ahumada-Fierro N.V., García-Mendoza E., Sandoval-Gil J.M., Band-Schmidt C.J. (2021). Photosynthesis and photoprotection characteristics related to ROS production in three *Chattonella* (Raphidophyceae) species. J. Phycol..

[60] Foyer C.H., Hanke G. (2022). ROS production and signalling in chloroplasts: Cornerstones and evolving concepts. Plant J..

[61] Boardman N.K. (2023). My journey to photosynthesis. Photosynth. Res..

[62] Reger B.J., Krauss R.W. (1970). The photosynthetic response to a shift in the chlorophyll *a* to chlorophyll *b* ratio of *Chlorella*. Plant Physiol..

[63] Dörmann P. (2006). Functional diversity of tocochromanols in plants. Planta.

[64] Amengual J. (2019). Bioactive Properties of Carotenoids in Human Health. Nutrients.

[65] Srivastava R. (2021). Physicochemical, antioxidant properties of carotenoids and its optoelectronic and interaction studies with chlorophyll pigments. Sci. Rep..

[66] Przybylska S., Tokarczyk G. (2022). Lycopene in the Prevention of Cardiovascular Diseases. Int. J. Mol. Sci..

[67] Rozanowska M., Edge R., Land E.J., Navaratnam S., Sarna T., Truscott T.G. (2019). Scavenging of Retinoid Cation Radicals by Urate, Trolox, and α-, β-, γ-, and δ-Tocopherols. Int. J. Mol. Sci..

[68] Singh R., Nesamma A.A., Narula A., Jutur P.P. (2022). Multi-Fold Enhancement of Tocopherol Yields Employing High CO_2_ Supplementation and Nitrate Limitation in Native Isolate *Monoraphidium* sp.. Cells.

[69] Sy C., Dangles O., Borel P., Caris-Veyrat C. (2015). Interactions between Carotenoids from Marine Bacteria and Other Micronutrients: Impact on Stability and Antioxidant Activity. Mar. Drugs.

[70] Stahl W., Heinrich U., Jungmann H., Sies H., Tronnier H. (2000). Carotenoids and carotenoids plus vitamin E protect against ultraviolet light-induced erythema in humans. Am. J. Clin. Nutr..

[71] Toyosaki T. (2002). Antioxidant effect of β-carotene on lipid peroxidation and synergism with tocopherol in an emulsified linoleic acid model system. Int. J. Food Sci. Nutr..

[72] Schroeder M.T., Becker E.M., Skibsted L.H. (2006). Molecular mechanism of antioxidant synergism of tocotrienols and carotenoids in palm oil. J. Agric. Food Chem..

[73] Kogure K. (2019). Novel Antioxidative Activity of Astaxanthin and Its Synergistic Effect with Vitamin E. J. Nutr. Sci. Vitaminol..

[74] Tesoriere L., Bongiorno A., Pintaudi A.M., D’Anna R., D’Arpa D., Livrea M.A. (1996). Synergistic interactions between vitamin A and vitamin E against lipid peroxidation in phosphatidylcholine liposomes. Arch. Biochem. Biophys..

[75] He M., Ding N.-Z. (2020). Plant unsaturated fatty acids: Multiple roles in stress response. Front. Plant Sci..

[76] Coniglio S., Shumskaya M., Vassiliou E. (2023). Unsaturated Fatty Acids and Their Immunomodulatory Properties. Biology.

[77] Srinivasan R., Mageswari A., Subramanian P., Suganthi C., Chaitanyakumar A., Aswini V., Gothandam K.M. (2018). Bicarbonate supplementation enhances growth and biochemical composition of *Dunaliella salina* V-101 by reducing oxidative stress induced during macronutrient deficit conditions. Sci. Rep..

[78] Maltseva I., Yakoviichuk A., Maltseva S., Cherkashina S., Kulikovskiy M., Maltsev Y. (2024). Biochemical and Antioxidant Characteristics of *Chlorococcum oleofaciens* (Chlorophyceae, Chlorophyta) under Various Cultivation Conditions. Plants.

[79] Ruzzi M., Sartori E., Moscatelli A., Khudyakov I.V., Turro N.J. (2013). Time-resolved EPR study of singlet oxygen in the gas phase. J. Phys. Chem. A.

[80] Guéraud F., Atalay M., Bresgen N., Cipak A., Eckl P.M., Huc L., Jouanin I., Siems W., Uchida K. (2010). Chemistry and biochemistry of lipid peroxidation products. Free Radic. Res..

[81] Li Y., Wei G., Chen J. (2004). Glutathione: A review on biotechnological production. Appl. Microbiol. Biotechnol..

[82] Gill S.S., Tuteja N. (2010). Reactive oxygen species and antioxidant machinery in abiotic stress tolerance in crop plants. Plant Physiol. Biochem..

[83] Portune K.J., Craig Cary S., Warner M.E. (2010). Antioxidant enzyme response and reactive oxygen species production in marine Raphidophytes. J. Phycol..

[84] Rezayian M., Niknam V., Ebrahimzadeh H. (2019). Oxidative damage and antioxidative system in algae. Toxicol. Rep..

[85] Schweitzer C., Schmidt R. (2003). Physical mechanisms of generation and deactivation of singlet oxygen. Chem. Rev..

[86] Triantaphylidès C., Krischke M., Hoeberichts F.A., Ksas B., Gresser G., Havaux M., Van Breusegem F., Mueller M.J. (2008). Singlet oxygen is the major reactive oxygen species involved in photooxidative damage to plants. Plant Physiol..

[87] Pereira L., Cotas J., Valado A. (2024). Antioxidants from microalgae and their potential impact on human well-being. Explor. Drug. Sci..

[88] De Carvalho C.C.C.R., Caramujo M.J. (2018). The Various Roles of Fatty Acids. Molecules.

[89] Casares D., Escribá P.V., Rosselló C.A. (2019). Membrane Lipid Composition: Effect on Membrane and Organelle Structure, Function and Compartmentalization and Therapeutic Avenues. Int. J. Mol. Sci..

[90] Galván I. (2017). Evidence of evolutionary optimization of fatty acid length and unsaturation. J. Evol. Biol..

[91] Bischoff H.W., Bold H.C. (1963). Phycological studies IV. Some Soil Algae from Enchanted Rock and Related Algal Species.

[92] Maltsev Y., Kezlya E., Maltseva S., Krivova Z., Ðinh C.N., Kulikovskiy M. (2025). Phylogeny and fatty acid profiles of new *Coccomyxa* (Chlorophyta) species from soils of Vietnam. Front. Microbiol..

[93] Zimmermann J., Jahn R., Gemeinholzer B. (2011). Barcoding diatoms: Evaluation of the V4 subregion on the 18S rRNA gene, including new primers and protocols. Org. Divers. Evol..

[94] White T.J., Bruns T., Lee S., Taylor J.W., Innis M.A., Gelfand D.H., Sninsky J.J., White T.J. (1990). Amplification and direct sequencing of fungal ribosomal RNA genes for phylogenetics. PCR Protocols: A Guide to Methods and Applications.

[95] Kumar S., Stecher G., Tamura K. (2016). MEGA7: Molecular evolutionary genetics analysis version 7.0 for bigger datasets. Molec. Biol. Evol..

[96] Drummond A.J., Rambaut A. (2007). BEAST: Bayesian evolutionary analysis by sampling trees. BMC Evol. Biol..

[97] Darriba D., Taboada G.L., Doallo R., Posada D. (2012). jModelTest 2: More models, new heuristics and parallel computing. Nature Meth..

[98] Stamatakis A., Hoover P., Rougemont J. (2008). A rapid bootstrap algorithm for the RAxML web–servers. Syst. Biol..

[99] Delgado-Zamarreño M.M., Bustamante-Rangel M., García-Jiménez M., Sánchez-Pérez A., Carabias-Martínez R. (2006). Off-line coupling of pressurized liquid extraction and LC/Ed for the determination of retinyl acetate and tocopherols in infant formulas. Talanta.

[100] Hossu A.-M., Radulescu C., Ilie M., Balalau D., Magearu V. (2006). Qualitative and semiquantitative TLC analysis of vitamins A, D and E. Revista Chim..

[101] Hamza T.A., Hadwan M.H. (2020). New spectrophotometric method for the assessment of catalase enzyme activity in biological tissues. Current Analytical. Chem..

[102] Sattar A.A., Matin A.A., Hadwan M.H., Hadwan A.M., Mohammed R.M. (2024). Rapid and effective protocol to measure glutathione peroxidase activity. Bull. Natl. Res. Cent..

[103] Sirota T.V. (2012). Use of nitro blue tetrazolium in the reaction of adrenaline autooxidation for the determination of superoxide dismutase activity. Biochem. Moscow Suppl. Ser. B.

[104] Maltseva S., Kezlya E., Krivova Z., Gusev E., Kulikovskiy M., Maltsev Y. (2022). Phylogeny and fatty acid profiles of *Aliinostoc vietnamicum* sp. nov. (Cyanobacteria) from the soils of Vietnam. J. Phycol..

[105] Kaszycki P., Walski T., Hachicho N., Heipieper H.J. (2013). Biostimulation by methanol enables the methylotrophic yeasts *Hansenula polymorpha* and *Trichosporon* sp. to reveal high formaldehyde biodegradation potential as well as to adapt to this toxic pollutant. Appl. Microbiol. Biotechnol..

